# Estimating the Underwater Diffuse Attenuation Coefficient with a Low-Cost Instrument: The KdUINO DIY Buoy

**DOI:** 10.3390/s16030373

**Published:** 2016-03-15

**Authors:** Raul Bardaji, Albert-Miquel Sánchez, Carine Simon, Marcel R. Wernand, Jaume Piera

**Affiliations:** 1 Department of Physical and Technological Oceanography, Marine Sciences Institute (ICM-CSIC), 37–49 Passeig Marítim de la Barceloneta, Barcelona E-08003, Spain; amsanchez@icm.csic.es (A.-M.S.); carine.simon@csic.es (C.S.); jpiera@icm.csci.es (J.P.); 2 Department of Physical Oceanography, Royal Netherlands Institute for Sea Research (NIOZ), PO Box 59. 1790AB Den Burg, Texel, The Netherlands; marcel.wernand@nioz.nl

**Keywords:** Arduino, buoy, citizen science, do-it-yourself, KdUINO, light, low-cost sensor, oceanography, diffuse attenuation coefficient

## Abstract

A critical parameter to assess the environmental status of water bodies is the transparency of the water, as it is strongly affected by different water quality related components (such as the presence of phytoplankton, organic matter and sediment concentrations). One parameter to assess the water transparency is the diffuse attenuation coefficient. However, the number of subsurface irradiance measurements obtained with conventional instrumentation is relatively low, due to instrument costs and the logistic requirements to provide regular and autonomous observations. In recent years, the citizen science concept has increased the number of environmental observations, both in time and space. The recent technological advances in embedded systems and sensors also enable volunteers (citizens) to create their own devices (known as Do-It-Yourself or DIY technologies). In this paper, a DIY instrument to measure irradiance at different depths and automatically calculate the diffuse attenuation *K_d_* coefficient is presented. The instrument, named KdUINO, is based on an encapsulated low-cost photonic sensor and Arduino (an open-hardware platform for the data acquisition). The whole instrument has been successfully operated and the data validated comparing the KdUINO measurements with the commercial instruments. Workshops have been organized with high school students to validate its feasibility.

## 1. Introduction

Water transparency is a common indicator to assess the environmental status of water bodies as it is strongly affected by different water quality components such as the concentration of phytoplankton or dissolved organic and inorganic components [1,2].

**Figure 1:**
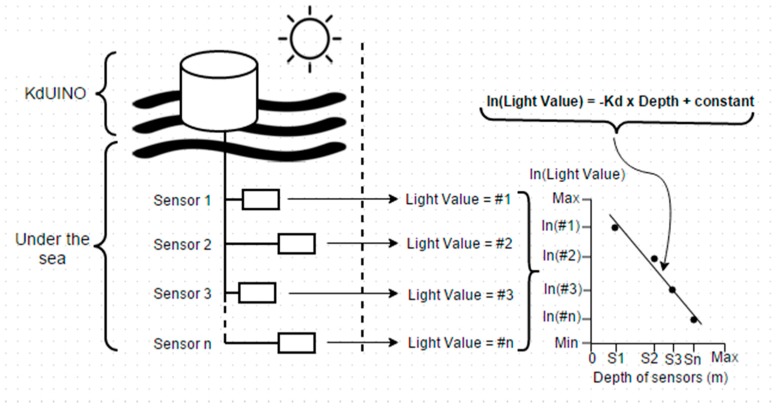
Overview of the KdUINO’s design and computation of $K_{d}$ as the slope of the linear regression of the measurements.

One parameter to quantify water transparency is the diffuse attenuation coefficient (*K_d_*), which is estimated by measuring the decrease of downwelling irradiance with depth. About 90% of the diffusely reflected light from the water column comes from a surface layer at a depth 1/*K_d_* [1], making *K_d_* a key parameter to correctly interpret remote sensing data. From the operational point of view, *K_d_* is a very practical parameter to use as it is computed from relative changes of irradiances (see Figure 1), and thus does not require the absolute radiometric calibration of sensors.

Recent large-coverage-high-resolution observations in coastal areas [3] and lakes [4,5,6] have shown that *K_d_* may have a high spatial heterogeneity and complex temporal dynamics. This mostly occurs in those areas with intense human activity and where natural factors such as sediment resuspension driven by wind and waves or river input are important. As an increasing number of coastal ocean field experiments shift from the shelf break toward the coast, sampling schemes must be adjusted to account for the more dynamic and complex physical processes, shorter temporal and spatial decorrelation scales, and more productive and turbid nearshore waters [7]. In order to study these complex systems, a high number of measurements in time and space have to be taken. Remote sensing via satellite measurements is a powerful tool to achieve good spatial coverage, however, in costal zones, shallow waters and small lakes, satellite data cannot be used because of the pixel size or the low frequency of the measurements. As noted by [8], “conventional satellites have a revisit time of around two days for most regions, which is further reduced if the area is frequently cloudy”. The Geostationary Ocean Color Imager is the only geostationary satellite able to give 8 measurements a day but its range of view is reduced to the Korean Peninsula. Therefore, to compensate this lack of data, *in situ* irradiance measurements are the only way to obtain the necessary information. But the number of such measurements obtained regularly with conventional instrumentation is relatively sparse in time and space, due to instrument costs and logistic requirements. The Secchi Disk is a classical citizen science instrument that allows one to measure the Secchi depth from which *K_d_* can be estimated, but the instrument produces low precision measurements, requires a strong human intervention (one trained person has to be present for each measurement) and only allows one to retrieve *K_d_* using empirical approximations of the measurement area. Besides, the retrieval of *K_d_* from the Secchi depth, even with approximations, is not well defined as demonstrated in [9].

In recent years, the promotion of volunteer monitoring programs, known as citizen science, has increased the number of environmental observations, both in time and space, and could be a potential solution to cover the gap of in-situ water quality observations.

One of the first requirements for monitoring water quality with a citizen science-based approach is to have affordable instruments for the volunteer observers. In this sense, several examples based on the development of new low-cost technologies have recently appeared for different kinds of optical instruments related to water quality. For instance, a novel turbidity meter suitable for basic water quality monitoring, which detects turbidity with range and precision similar to expensive commercial instruments, is presented in [10] a cost effective *in situ* fluorometer is described in [11], or a signal processing chain to process the fluorescence spectra of marine organisms for taxonomic discrimination for low-cost instruments is introduced in [12]. However, none of these techniques are specifically designed to estimate *K_d_*.

In this paper, we present the design of the KdUINO, a low-cost moored system that measures downwelling irradiance at different depths with *K_d_* as an output parameter. Data-acquisition is based on an open-source hardware platform (Arduino, [13]) that controls and stores the data obtained from several quasi-digital optical sensors placed at different depths. Some post-processing analysis allows to retrieve the *K_d_* parameter by using the relative irradiance values obtained from the different sensors. In addition, the choice of quasi-digital sensors (light-frequency converters) and instrumental design minimizes the measurement errors. Using a suitable averaging process, the KdUINO can provide reliable measurements even in the first metres of the water column, where *K_d_* estimations are usually discarded as they are affected by the focusing and defocusing of direct sunlight after different refractions of the sun rays on the water surface [14].

The simple structure of the system combined with the low-cost electronics (under 100 US dollars) converts this instrument into a suitable Do-It-Yourself (DIY) tool available for a citizen science-based water quality monitoring. The instrument is available as well for research laboratories and environmental monitoring companies with the need of cost-effective *K_d_* estimations with high spatial and temporal resolution.

The KdUINO is thus an appropriate instrument to overcome the weaknesses of existing *in situ* instrumentation and to cover the gap of temporal coverage left by satellite data. The construction of the KdUINO system and, details of the sensors, along with their optical characterization and performance are described below. The validation of the buoy with commercial instruments is also presented.

## 2. Design and Manufacturing of the KdUINO

The design of the KdUINO has been made as simple as possible in order to allow its implementation by scientists or volunteers with only basic electronic skills. This section describes the principles the KdUINO is based on and details the different steps of its design.

### 2.1. KdUINO Principles

The Beer-Lambert Law relates the absorption of light to the properties of the medium through which the light is traveling. In particular, when applied to a liquid medium, it states that the light intensity decreases exponentially as a function of depth which is mathematically written as:

(1) $$E_{2}=E_{1}e^{-K_{d}\Delta z}$$

where $E_{2}$ is the irradiance of the light at depth $z_{2}$, $E_{1}$ is the irradiance of the light at depth $z_{1}$, and $\Delta z=z_{2}-z_{1}$ is the difference of depth in metres. $E_{1}$ and $E_{2}$ are wavelength dependent, but, here and in the rest of the manuscript, its dependence is dropped for simplicity. Therefore, *K_d_* is calculated as the slope of the straight line obtained by applying the natural logarithm on Equation (1):

(2) $$lnE_{2}=-K_{d}\Delta z+ln\left(E_{1}\right)$$

If $E_{1}$ is the irradiance of the light very near the surface (*i.e.,* at $z_{1}\;\sim \;0$), it is possible to extract the irradiance of the light at any depth $z_{i}$ in the water column as follows:

(3) $$lnE_{i}=-K_{d}\Delta z+constant$$

where $constant=\ln\left(E_{1}\right)$.

Thus, in order to estimate *K_d_*, the KdUINO measures the light intensity at several depths in the water column using several low-cost sensors. As illustrated in Figure 1, using the linear regression of a set of such measurements, $K_{d}$ is easily retrieved as the negative of the slope of the linear regression.

To get a robust estimation of the attenuation coefficient, the KdUINO works as follows: it saves the measurements in an internal memory; a quick analysis of the data provides information on the trend of *K_d_*. In the event of an outlier, if it has a low coefficient of determination, it is noted as “erroneous data”. The coefficient of determination of the linear regression (*r*^2^) is used to indicate how well the data of the KdUINO fits to the line with a slope of −*K_d_*. A threshold is chosen for *r*^2^ and data which correspond to a lower value are rejected. When the KdUINO is used in the laboratory or in lakes with no waves, the selected threshold for *r*^2^ is of around *r*^2^ > 0.95 but in the sea, due to the waves and the continuous movement of the buoy, the threshold has to be lowered to around *r*^2^ > 0.75. Lower values of *r*^2^ can be due to the malfunction of one of the sensors or to measurements in layers with different transparency (for instance, above and below a thermocline). In case of damaged sensors, the software, provided in the supplementary material, automatically discards its measurements and the estimation of *K_d_* is carried out with the remaining sensors (as long as the number of remaining sensors is greater than or equal to three). If sensors are placed in different transparency layers, users can estimate *K_d_* with the same methodology in each layer separately as long as there are three sensors in each layer. Although the KdUINO does not need absolute calibration to calculate$\;K_{d}$, Equation (3), the sensors do need to be inter-calibrated, as explained in Section 4.

### 2.2. KdUINO Components

**Figure 2:**
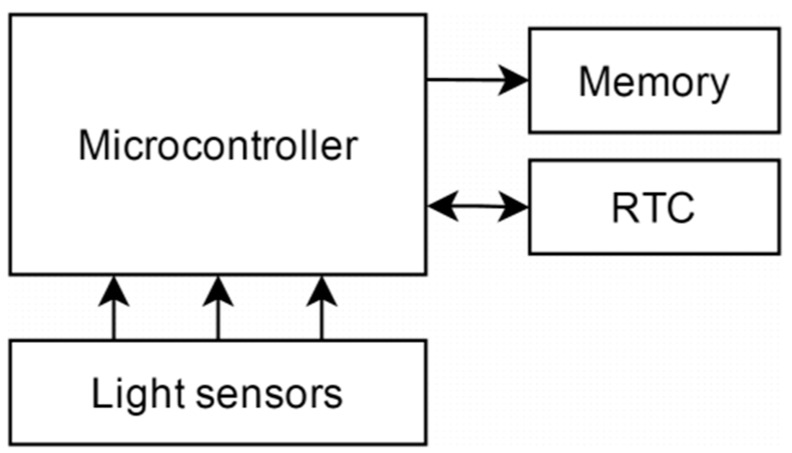
Basic electronic schema.

The design of the buoy follows the concept of DIY technology, with inexpensive and easy-to-use components. Figure 2 presents a general schema of the different electronic parts of the KdUINO. A limited number of sensors (minimum three, maximum six) are used to measure the subsurface irradiance and the microcontroller receives the data from the sensors and stores it in the memory. Included in the system is a real time clock (RTC) to establish the measurement time.

**Figure 3:**
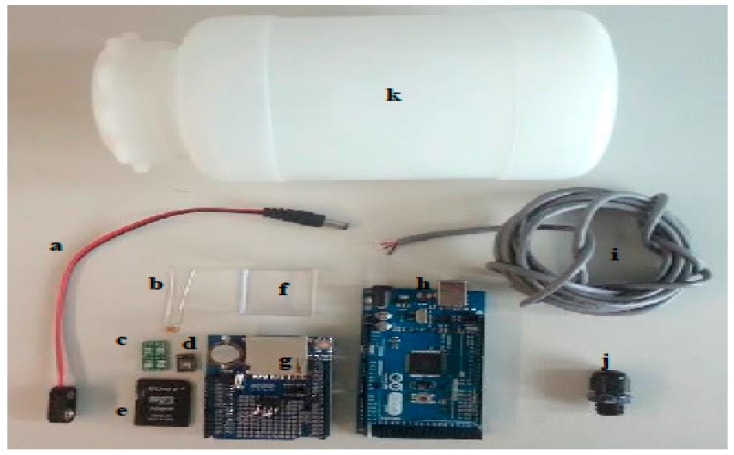
KdUINO components, (**a**) 9 V Battery button power plug for Arduino; (**b**) 100 nF Capacitor; (**c**) 8 pin SOIC to DIP8 Adapter; (**d**) SD memory card; (**e**) TSL230RP; (**f**) Polyester transparent box; (**g**) Data Logger Module Logging Recorder Shield V1.0; (**h**) Arduino MEGA 2560 R3; (**i**) Industrial cable, 3 cores; (**j**) Cable Gland Nylon 66; (**k**) Hermetic bottle.

Besides the electronics, silicon paste, a bottle and boxes to house the electronics are needed. Table 1 lists the prices of all the components needed to make the instrument. Taxes, shipping and handling costs are not included. Figure 3 illustrates all the necessary components priced in Table 1.

**Figure 4:**
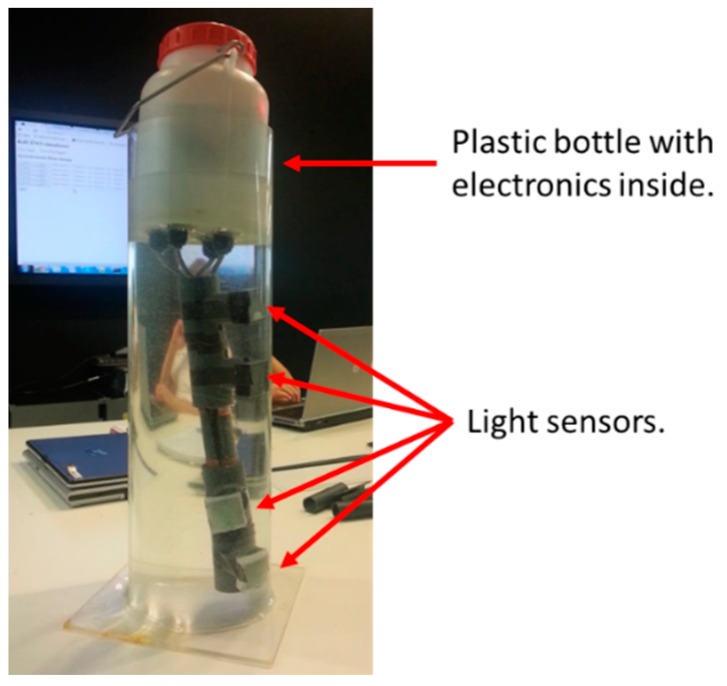
Presentation of the KdUINO.

Figure 4 shows the whole KdUINO after being implemented inside a transparent vertical tube with water. Electronics are inside the plastic bottle and the four light sensors were attached to a PVC pipe to control their position and orientation in the water column (see laboratory set-up of Figure 4).

### 2.3. KdUINO Firmware and Software

A firmware is used with the microcontroller Arduino Mega to count the number of pulses received from each sensor during a fixed period of time. Then, the number of pulses of each sensor and the date and time obtained from the RTC are stored in the SD memory card with the name of “datalog.txt”. This process is continuously performed while the electronics are switched on. The firmware, provided in the supplementary material as “KdUINO_Sensors.ino”, has been developed using the programming framework Wiring with the Arduino IDE v1.5.8. The code needs the additional open-source libraries “SD-master” and “RTClib-master” for the management of the system data logger. They can be downloaded from the web page of the manufacturer [16].

To determine *K_d_*, the calibration (see Section 4) and data files from the SD card must be downloaded to a computer. This information is post-processed with a software code developed under Python v2.7. The code first computes the calibration factor (see Section 4) and applies it to all the measured values. Finally, the code uses the Beer-Lambert Law Equation (3) and the linear regression to obtain *K_d_*. The software, which can be found in the supplementary material as “KdUINO_analysis.py”, uses the open-source libraries “numpy” [17], “matplotlib” [18] and “scipy” [19].

### 2.4. Building of A KdUINO

The manufacturing of the KdUINO is divided in two parts; (1) assembly of the light sensors and (2) connections.

#### 2.4.1. Implementation of the Light Sensors

The light sensors used in the KdUINO are so-called quasi digital-optical sensors and convert light intensity into a frequency signal (square waves). This makes the direct use of the digital ports of the microcontroller possible, avoiding any analog-to-digital converters. Light-to-frequency converters have an additional advantage: they allow long time integrated measurements by simply counting the number of pulses, hence obtaining an average from several seconds to minutes. Large time integrations are important to mitigate light variability in the water column caused by the surface wave effect and to correctly estimate the water transparency [14]. This type of sensors has thus been chosen and, in particular, the TSL230RP [15], because of its low cost, linearity and easy operation.

The TAOS light sensor has four configuration pins (S0, S1, S2 and S3), which allows configuring the reception sensitivity and the scaling of the output frequency (see datasheet TSL230RP [15]). The sensitivity configuration of the sensor must be modified according to the amount of light intensity that users need to measure. The output frequency can be scaled by (*i.e.*, divided by) 1, 2, 10 or 100 depending on the users’ configuration. The measurements obtained in several experiments have determined that the optimal configuration for using the sensors in coastal waters is:

- S0 = 0, S1 = 1: Configuration of the sensor’s sensitivity at middle level.
- S2 = 1, S3 = 1: Configuration of the output frequency scaling at 100.

**Figure 5:**
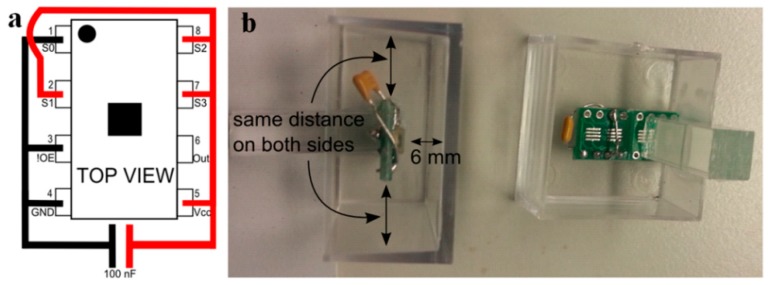
(**a**) Electrical schema of the light sensor; (**b**) Position of the sensor in transparent polyester box.

Figure 5a shows the pin configuration of the sensor’s integrated circuit. Pins 4 (GND) and 5 (V_CC_) are interconnected through a 100 nF capacitor. Pins 2 (S1), 7 (S3) and 8 (S2) are connected to pin 5 (V_CC_). Pin 1 and 3 (!OE) are connected to pin 4 (GND). A small green eight pin SOIC to DIP8 Adapter Prototype Circuit Board (PCB) was used to solder the sensor and its connections.

Once the sensor soldered, it is placed in a transparent polyester box, as seen in Figure 3b. The position of the sensor inside the box should respect the following considerations:

- The sensor must point to the base of the box.
- The sensor must be placed parallel to the base of the box.
- The sensor must be placed at the center of the box.

One way to fulfill these requirements is to glue the sensor to a small methacrylate stick which is in turn glued to the box, see Figure 3b.

#### 2.4.2. Connection of the Sensor to the Cable

**Figure 6:**
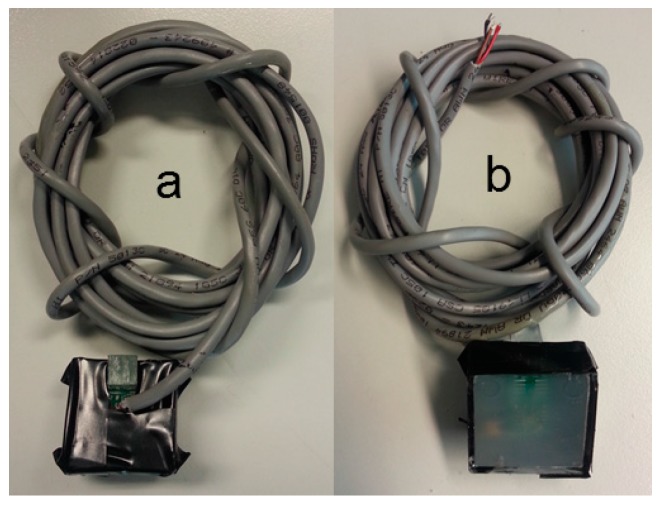
(**a**) Back of the sensor; (**b**) Front of the sensor.

Once the sensors have been placed in their transparent polyester boxes, the V_CC_ wire is soldered to pin 5, the GND wire to pin 4 and the data wire to pin 6 (with the other end connected to the microcontroller external interruption port). Finally, after checking the sensor’s performance, some Synolite resin is poured into the box completely covering the sensor and cable’s junctions and making them fully waterproof. Once the resin has dried, a black tape is used to cover the sides and the top of the box (*i.e.*, the opposite side of optical sensor’s orientation), Figure 6, so the light only enters into the sensor’s box through its base.

#### 2.4.3. Connection of Electronics

The core of the buoy is an Arduino board, a platform commonly used in DIY technology projects (it is open-source and open-hardware). The Arduino systems provide some sets of digital and analog I/O pins that can be interfaced to various extension boards or sensors, a microcontroller and serial communication interfaces to load the firmware. Among the different possibilities, the model boards needed to develop the KdUINO are:

- The Arduino board model Mega (Arduino), because it is the only one with six external interruption pins to connect the optical sensors.
- The Data Logging Shield V1.0 preassembled board (Earl, Adafruit Data Logger Shield, 2015), which includes an RTC to know the date and time of the instrument measurements and an SD memory card adapter that allows saving the data in an SD card. This board is plugged on the Arduino main board.

**Figure 7:**
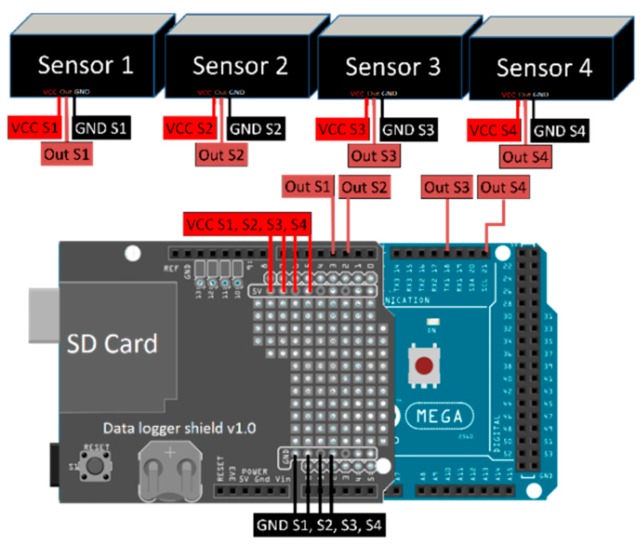
Connection of sensors, the Data Logger Shield v1.0 and the Arduino MEGA.

Figure 7 shows the connections of all the electronic devices of the KdUINO, with the Data Logging Shield plugged on top of the Arduino Mega board. The light sensors are connected to different pins of the Arduino Mega (D3, D2, D18 and D19 for a four-sensor buoy; if it is extended to six sensors, D2 and D3 should be used as well) using special waterproof cables. Finally, an SD memory card and a CR1220 button battery are placed in the specific adapters of the Data Logging Shield.

The Arduino board is powered by a 9-Volt battery which is enough to make the electronics work for about 6 h and 20 min. More powerful batteries can be used if necessary. The Arduino Mega and the Data Logger Shield are placed in the hermetic plastic bottle (see Figure 4). Sensors are outside the bottle but connected to the Arduino Mega with cables. Holes are made in the hermetic bottle to pass the sensor cables, and must be carefully sealed with silicone to prevent water dripping inside the bottle.

## 3. Characterization of the Sensors

In underwater optical measurements two kinds of irradiance collectors are used, scalar and cosine. The cosine detector proposed here is a vector irradiance sensor with a directional response proportional to the cosine of the relative zenith angle of incidence, usually with the shape of a button. The cosine irradiance is abbreviated as *E_d_* or *E_u_* for downwelling or upwelling orientations respectively. As *K_d_* must be obtained from *E_d_* measurements, the KdUINO sensors can only be implemented using cosine detectors.

The TAOS TLS230RP light sensor, used in the KdUINO buoy, has a spectral range which is accurately known, provided by the manufacturer [15]. However, because the light sensor is fully encapsulated in Synolyte to make it waterproof, a re-characterization is necessary. The characterization setup and measurement results are described below.

### 3.1. Darkroom Setup

**Figure 8:**
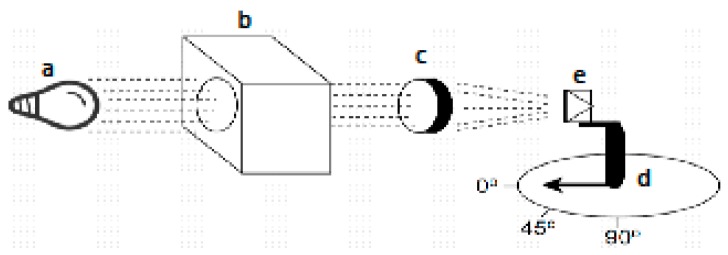
Schema of the instrumentation in the black-room laboratory. (**a**) Horizontal quartz-halogen standard lamp; (**b**) Zeiss spectral monochromatic; (**c**) spherical lenses; (**d**) mechanical platform with focus variable angle; (**e**) the sensor (installed on a rotating platform).

The measurements of the TLS230RP sensors placed in Synolyte capsules were performed in a darkroom laboratory equipped with a Horizontal quartz-halogen standard lamp, a Zeiss spectral monochromator, two spherical lenses and a mechanical platform to install the sensors at a desired angle with respect to the light beam (see Figure 8).

The setup of Figure 8 was firstly used to obtain the spectral response of the sensors. For these measurements, the TLS230RP sensors in Synolyte capsules were fixed pointing to the light beam generated by the quartz-halogen lamp. Using the Zeiss spectral monochromatic filters, measurements were performed between 350 and 950 nm in steps of 50 nm and using an average of ten seconds per measurement. In this case, the angle between the sensors and the light beam staid constant at 0°.

The same setup, this time without the Zeiss spectral monochromatic filter, was secondly used to characterize the new directivity of the optical sensors. For this setup, the angle of the TLS230RP sensors with respect to the light beam was increased from 0° to 90° in steps of 10°, and each measurement was obtained again using a 10 s average.

#### 3.1.1. Spectral Characterization

**Figure 9:**
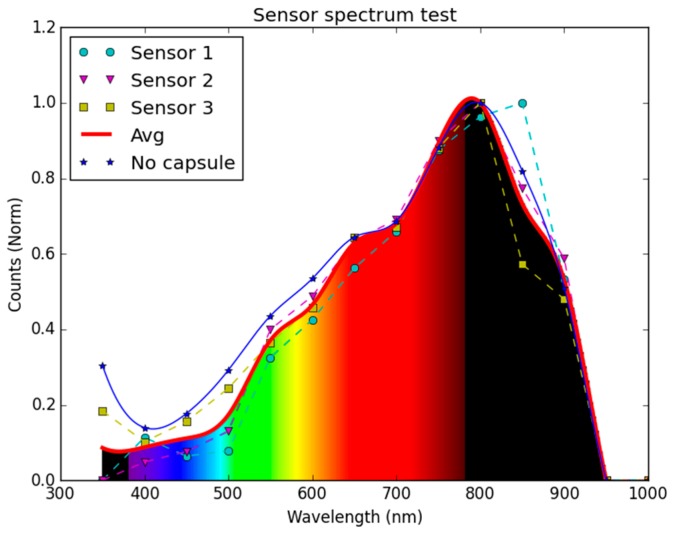
Spectrum response of the TSL230RD with the Synolite capsule and comparison to the response without capsule.

Figure 9 shows the spectral response of the three different sensors. Although slightly different amounts of Synolite were present over each sensor due to imperfect implementations, a similar spectral sensitivity was obtained in the three cases. As can be seen in Figure 9, their behavior is also equivalent to a non-encapsulated sensor. The measurements indicate that the sensors are able to detect wavelengths between 350 nm and 950 nm, with a maximum sensitivity around 800 nm. The largest differences were found at 500 and 850 nm. Again, these results are similar to the spectral response of the TLS230RP provided by the manufacturer [15], showing that the Synolite capsule has little effect on their optical performance. It must be noted that the sensitivity curve is not constant in the traditional Photosynthetically Active Radiation (PAR) region (400 to 700 nm) and, besides, it extends into the near infrared region. However, water presents a strong light attenuation in the whole Infra-Red (IR) range [20] and a high attenuation over the blue wavelengths. The combination of this natural effect with the sensor’s sensitivity curve generates the approximate response of a PAR region filter, smoothing the spectral response shape between 400 and 700 nm and filtering the contribution beyond this bandwidth (*i.e.,* between 350–400 nm and 700–900 nm). Therefore, the estimations of *K_d_* obtained from measurements with the KdUINO are strongly related to the integrated PAR values. Furthermore, applying Equation (4), the correlation is even better. In some very specific cases, like in very turbid water conditions, the correlation with *K_d_* PAR could be lower because of the increase of *K_d_* in the band of NIR. Adding an NIR block filter on top of the transparent box of the sensor, the error caused by this effect would be reduced. However, in all the measurements we made (see Section 5.2 below), we did not detect any non-negligible error affecting the correlation with *K_d_* PAR. In the following, *K_d_* will stand for *K_d_* PAR.

To conclude this section, the analysis of directivity and spectral range results indicate that the performance of the TLS230RP sensors with and without the Synolite capsule is very similar, so the encapsulated sensors can indeed be used for *K_d_* estimations.

#### 3.1.2. Cosine Characterization

**Figure 10:**
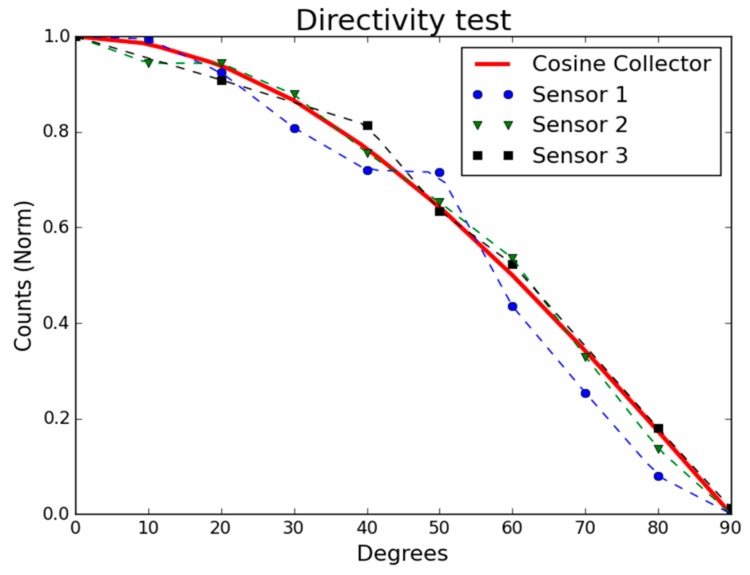
Comparison of measurements obtained with three different TLS230RP sensors encapsulated in Synolite and the ideal response of a cosine collector.

Figure 10 shows the measured directivity of the three sensors and the ideal response of a cosine collector for comparison purposes. As can be seen, the three sensors respond similarly to the ideal cosine collector.

## 4. Calibration of Sensors

As already noted above, *K_d_* is computed using relative measurements from the sensors placed at different depths in the water column, *i.e.*, it is not necessary to know the actual value of the light intensity at each point in the water column, but only the relative decrease of one sensor value with respect to the sensor above. It is therefore essential that all the sensors provide exactly the same frequency value when measuring the same light intensity. However, due to an absolute frequency tolerance of ±20% from the manufacturing procedure of the TSL230RD [15] and to a possible slight difference in their position and orientation in the transparent polyester box, different sensors under the same measuring conditions provide different measurement results by default. In order to compensate such differences, they must be calibrated. The calibration consists in measuring the same light intensity during the same period of time simultaneously using all the sensors of a single buoy and then compensate the difference as follows: the sensor with the highest measurement value is taken as the reference sensor. A multiplying factor is then computed for the other sensors to get the same intensity value as the one obtained by the reference sensor under the same conditions.

**Figure 11:**
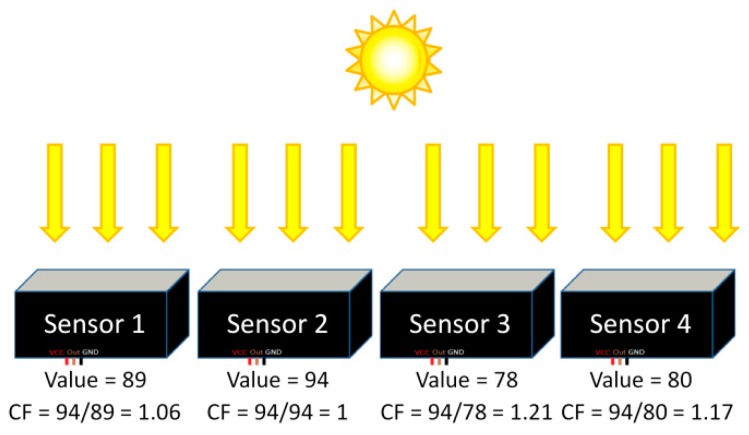
Example of sensor calibration where the Calibration Factor (CF) of each sensor is calculated.

The firmware of the Arduino Mega that performs the calibration of the sensors (found in the supplementary material as “KdUINO_calibration.ino”) counts the number of pulses provided by each optical sensor in one minute. The number of pulses of each sensor is saved in the SD memory card. Figure 11 shows an example of the process for the sensor calibration using the firmware. After placing the four sensors facing the sun, and thus, receiving the same light intensity, each sensor provides a measurement value different from the other three. In this case, since sensor 2 presents the highest value (94), it is taken as the reference sensor. The value of each sensor is then scaled according the reference sensor value. For sensor 1, the calibration factor is 1.06; for sensor 3 it is 1.21; and for sensor 4 it is 1.17. So for all succeeding measurements, the values obtained with the different sensors will be compensated by these calibration factors which will be stored in the SD memory card.

The calibration should be done every time that the user changes or adds a new sensor. It is also recommended to recalibrate the sensors from time to time, for example, whenever the sensors are cleaned to avoid the biofouling. It is important to note that sensors must not be placed under a shadow, neither for the calibration nor for the real measurements.

The calibration factors are included in the processing code that estimates *K_d_*. After calibration, the KdUINO is ready for use. Operators should place each sensor at a different (known) depth with the sensor looking up. Attention should be paid when placing the buoy in order to avoid overshadowing any sensor. A rigid pipe or a stick can be useful to fix the sensors in the correct position, as shown in Figure 4. The algorithm to calculate *K_d_* needs as input the operating water depth of each optical sensor in metres, therefore, users must manually measure these distances and include the operating water depth values of each sensor in the processing code.

## 5. Experimental Measurements

In this section the validation measurements of the KdUINO are presented by comparing its results with ones obtained with classical oceanographic instruments. An experiment of construction and use by laymen is also explained.

### 5.1. Validation of KdUINO Measurements of K_d_ PAR in Laboratory Conditions

In order to validate the KdUINO measurements, the *K_d_* *PAR* estimated from the analysis of its measured data was compared with the *K_d_* *PAR* obtained using commercial scientific instruments as the PRR-800 [21] and the RAMSES-ACC-VIS [22]. The PRR-800 is a high resolution profiling reflectance radiometer and its cost is over 30,000 US dollars. The RAMSES-ACC-VIS is a stand-alone highly integrated hyperspectral radiometer for the UV and/or VIS spectral range and its cost is over 10,000 US dollars. Both can measure the irradiance of the light at several wavelengths and can also estimate the PAR.

#### 5.1.1. Setup

**Figure 12:**
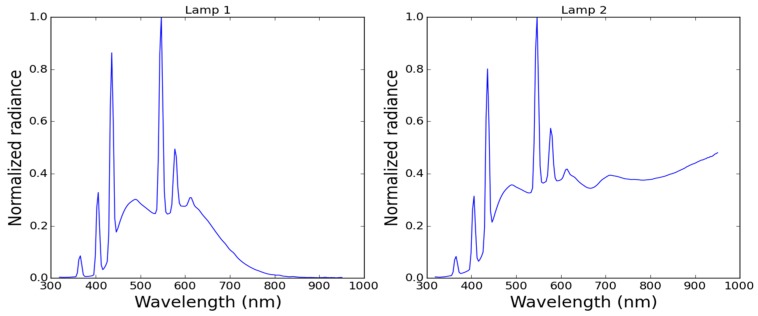
Lamp spectra of the experimental tank.

A first set of measurements was made using the RAMSES and the KdUINO in an experimental tank of 3 m depth placed in a laboratory. Two different lamps were used to simulate different kind of sun radiation. Figure 12 shows the spectra response of the two radiation lamps. As can be observed, the main difference is that the second lamp has an important emission in the infrared region while the first lamp has almost no emission above 800 nm.

Another set of measurements was performed in shallow coastal areas. The weather conditions during the field measurements were stable and with small waves (less than 30 cm). In this case, the KdUINO was used along with the PRR-800 and the RAMSES. To estimate *K_d_* with the radiometers, several irradiation measurements were taken at each depth of the water column. Then, an average of the measurements at each depth was calculated in order to minimize the possible variation of light detected by the sensors produced by the surface waves. In order to compare the results of the commercial oceanographic instruments and the KdUINOs, different KdUINOs have been used during the experimental measurements.

#### 5.1.2. Measurement Results

**Figure 13:**
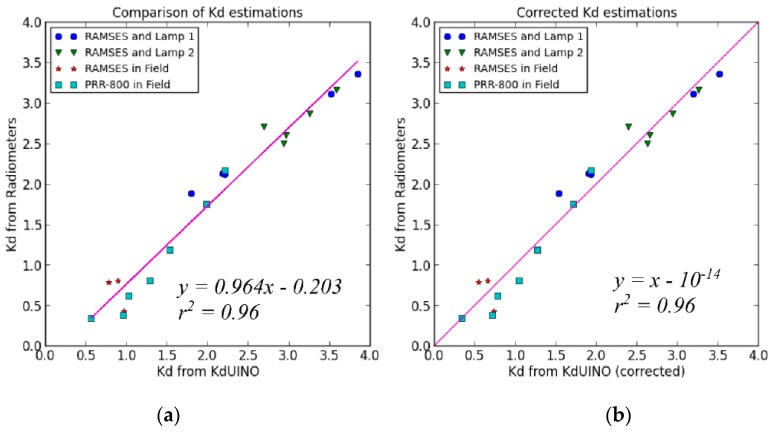
(**a**) Comparison of *K_d_* results derived from commercial instruments: the hyper-spectral RAMSES radiometer and the PRR-800, and the KdUINO; (**b**) Same comparison after spectral response compensation of the original KdUINO measurements (see text).

Figure 13a compares the estimation of *K_d_* PAR obtained using the oceanographic radiometers and the KdUINO. The plots combine the tank and the field measurements realized with several KdUINOs and various reference instruments. A strong linear relationship can be seen between the estimations of the different KdUINO measurements and those of the reference instruments (Figure 13a). The linear regression produces a line with a slope of 0.964, and a *y*-intercept of −0.2 with *r*^2^ of 0.96. The initial bias observed in this plot can be associated with the fact that the spectral response of the KdUINO sensors (Figure 9) has not been corrected to estimate PAR (a normal procedure in commercial instruments). This bias can be easily compensated by applying the linear transformation shown in Equation (4), where the coefficients were derived from the initial comparison (Figure 13a). Figure 13b compares the corrected estimations of *K_d_*, based on KdUINO measurements, with the ones of the commercial radiometers, showing the final unbiased linear agreement:

(4) $$K_{d}\left(corrected\right)=0.964K_{d}\left(original\right)-0.203$$

### 5.2. Testing DIY Construction and Deployment under Field Conditions

**Figure 14:**
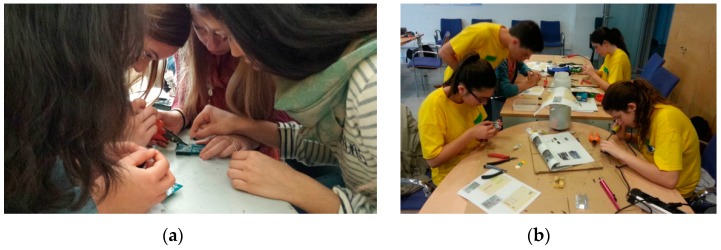
Students from Sant Carles de la Rapita (**a**) and Mollet (**b**) building their own KdUINOs.

In order to validate the overall concept of KdUINO as a DIY citizen science instrument, several workshops were offered in high schools to test if students were able to build the buoys on their own, following the step-by-step tutorials developed for this purpose. Figure 14 illustrates the strong motivation of all the volunteers who were able to construct the KdUINOs. At the end of the workshops, two different locations were selected to test the buoys, one in Barcelona and the other one in Alfacs Bay (in the Ebro delta, Spain). Alfacs Bay area is much richer in phytoplankton than the rest of the Catalan coast (including the Barcelona beach), so the transparency of the water is usually lower than in the other zones. Consequently, *K_d_* in Alfacs Bay is usually higher than in the Barcelona beach.

**Figure 15:**
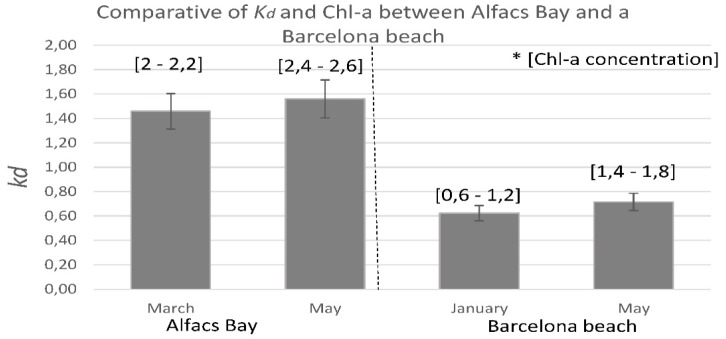
Results of the analysis of data in the Barcelona beach and the Alfacs Bay, data in square brackets correspond to the chlorophyll concentration ranges recorded in previous studies [23,24]. It can be seen that higher levels of chlorophyll concentration lead to higher attenuation coefficient.

The results presented in Figure 15 show the *K_d_* values obtained with the different buoys built by the students. The obtained *K_d_* estimations are in coherence with the expected values, taking as a reference the ranges of chlorophyll concentrations previously recorded in both sites at those particular months [23,24]. Although further field test are obviously necessary, these preliminary experiments are sufficient to demonstrate the goal of the present study: the proof of concept that the buoy is a device that can be built by volunteers (*i.e.*, it is a real DIY instrument), and that may provide, at the same time, valuable field data.

## 6. Conclusions

This study presents a low-cost DIY buoy to measure the diffuse attenuation coefficient *K_d_* of PAR that can be easily assembled by citizens. The buoy consists of some quasi digital-optical light sensors to collect the data and an electronic platform with a Real Time Clock and an SD memory card to save the data and the time when the data has been taken. Since the KdUINO has been designed to be simple and modular, optical sensors can be added or extracted depending on the application and the compromise between the price, the simplicity and the use, considering that at least three optical sensors are needed to properly estimate *K_d_*.

In this paper, the optical sensors used for KdUINO were spectrally and directionally characterized and some tests have proven that the buoy system generated satisfactory results, *i.e.*, comparable with results obtained from professional radiometers.

The used light intensity-to-frequency converter sensors have the ability to perform large time integrations, which is important to average light variability in the sea caused by the surface wave effects.

The price of a KdUINO is around 100 US dollars, at least 10 times less than a classical PAR instrument, so for a fixed budget, a much denser spatio-temporal coverage of areas of interest is possible. This allows a high quality water monitoring of coastal waters, lakes and estuaries, taking into account the big variability in space and time of the water transparency in these areas. The field results obtained in the proof of concept experiment were satisfactory. With this first achievement, it is now possible to start designing new field experiments to evaluate the full potential of the KdUINO in retrieving water quality derived parameters.

## Figures and Tables

**Table 1:** Supplies including prices in US dollars (2015) to build a KdUINO.

Component	Source	Price Per Unit
1 × Arduino MEGA 2560 R3 [13]	www.aliexpress.com	$9.80
4 × TSL230RP [15]	www.es.rs-online.com (store ref. 642–4395)	$3.31
4 × 100 nF Capacitor	www.es.rs-online.com (store ref. 699–4891)	$0.44
4 × 8 pin SOIC to DIP8 Adapter	www.aliexpress.com	$0.08
132 mL × Synolite and catalyst	www.drogueriaboter.es	$19.72 (1 L)
6.4 m × Industrial cable, 3 cores	www.es.rs-online.com (store ref. 168–0146)	$1.45/m
4 × Polyester transparent box (29 mm × 29 mm × 15 mm)	www.servicioestacion.es	$0.63
1 × Data Logger Module Logging Recorder Shield V1.0 (Earl, Adafruit Data Logger Shield, 2015)	www.aliexpress.com	$5.45
1 × 9 V Battery button power plug for Arduino	www.aliexpress.com	$2.35 (2 units)
1 × SD memory card (8 GBytes)	www.aliexpress.com	$5.63
1 × Hermetic bottle	www.servicioestacion.es	$6
4 × Cable Gland Nylon 66, IP68, M12 × 1.25	www.es.rs-online.com (store ref. 669–4654)	$3.47 (5 units)
**Total in US dollars**		$60.57

## Supplementary Materials

The Arduino Firmware, the Python code for data analysis and some examples of files generated with a KdUINO are available online at http://www.mdpi.com/1424-8220/16/3/373/s1.

## References

[1] Mobley D. (1994). Optical Properties of Water. Light and Water: Radiative Transfer in Natural Waters.

[2] Sosik M., Babin M., Roesler C.S., Cullen J.J. (2008). Characterizing Seawater Constituents from Optical Properties. Real-time Coastal Observing Systems for Ecosystem Dynamics and Harmful Algal Blooms.

[3] Mishra D.R., Narumalani S., Rundquist D., Lawson M. (2005). Characterizing the vertical diffuse attenuation coefficient for downwelling irradiance in coastal waters: Implications for water penetration by high resolution satellite data. ISPRS J. Photogramm. Remote Sens..

[4] Shi K., Zhang Y., Liu X., Wang M., Qin B. (2014). Remote sensing of diffuse attenuation coefficient of photosynthetically active radiation in Lake Taihu using MERIS data. Remote Sens. Environ..

[5] Zhang Y., Zhang B., Ma R., Feng S., Le C. (2007). Optically active substances and their contributions to the underwater light climate in Lake Taihu, a large shallow lake in China. Fundam. Appl. Limnol..

[6] Liu X., Zhang Y., Yin Y., Wang M., Qin B. (2013). Wind and submerged aquatic vegetation influence bio-optical properties in large shallow Lake Taihu, China. J. Geophys. Res. Oceans.

[7] Chang G.C., Dickey T.D., Schofield O.M., Weidemann A.D., Boss E., Pegau W.S., Moline M.A., Glenn M.A. (2002). Nearshore physical processes and bio-optical properties in the New York Bight. J. Geophys. Res. Oceans.

[8] Lee Z., Jiang M., Davis C., Pahlevan N., Ahn Y., Ma R. (2012). Impact of multiple satellite ocean color samplings in a day on assessing phytoplankton dynamics. Ocean Sci. J..

[9] Lee Z., Shang B., Hu C., Du K., Weidemann A., Hou W., Lin J., Gong L. (2015). Secchi disk depth: A new theory and mechanistic model for underwater visibility. Remote Sens. Environ..

[10] Kelley C.D., Krolick A., Brunner L., Burklund A., Kahn D., Ball W.P., Weber-Shirk M. (2014). An Affordable Open-Source Turbidimeter. Sensors.

[11] Leeuw T., Boss E.S., Wright D.L. (2013). *In situ* Measurements of Phytoplankton Fluorescence Using Low Cost Electronics. Sensors.

[12] Aymerich I.F., Sánchez A.-M., Pérez S., Piera J. (2015). Analysis of Discrimination Techniques for Low-Cost Narrow-Band Spectrofluorometers. Sensors.

[13] Arduino Mega. http://arduino.cc/en/Main/arduinoBoardMega.

[14] Darecki M., Stramski D., Sokólski M. (2011). Measurements of high-frequency light fluctuations induced by sea surface waves with an Underwater Porcupine Radiometer System. J. Geophys. Res..

[15] TAOS TSL230RD, TSL230ARD, TSL230BRD Programmable Light-to-Frequency Converters. TAOS054P Datasheet, 2007. http://www.datasheetlib.com/datasheet/1151779/tsl230ard_taos-texas-advanced-optoelectronic-solutions.html.

[16] Adafruit Data Logger Shield. https://learn.adafruit.com/adafruit-data-logger-shield.

[17] Numpy. http://www.numpy.org/.

[18] Matplotlib. http://matplotlib.org/.

[19] Scipy. http://www.scipy.org/.

[20] Pegau W.S., Gray D., Ronald W.S., Zaneveld V. (1997). Absorption and attenuation of visible and near-infrared light in water: Dependence on temperature and salinity. Appl. Opt..

[21] Biospherical Instruments Inc. PRR-800 Profiling Reflectance Radiometer. http://www.biospherical.com/BSI%20PDFs/Brochures/prr-800.pdf.

[22] TriOs Optical Sensors RAMSES-ACC-VIS Hyperspectral UV-VIS Irradiance Sensor. http://www.rshydro.ie/resdev/Ramses-ACC-VIS-pr-629.html.

[23] Fernández M., Castan V., Dàmaso E. (2015). Report of Toxic Phytoplankton Monitoring and Environmental Parameters in Ebro Delta Bays.

[24] Arin L., Guillén J., Segura-Noguera M., Estrada M. (2013). Open sea hydrographic forcing of nutrient and phytoplankton dynamics in a Mediterranean coastal ecosystem. Estuar. Coast. Shelf Sci..

